# Ketogenic diet with aerobic exercise can induce fat browning: potential roles of β-hydroxybutyrate

**DOI:** 10.3389/fnut.2024.1443483

**Published:** 2024-08-29

**Authors:** Sujin Kim, Dong-Ho Park, Sohee Moon, Bonsang Gu, Keren Esther Kristina Mantik, Hyo-Bum Kwak, Ji-Kan Ryu, Ju-Hee Kang

**Affiliations:** ^1^Department of Pharmacology, College of Medicine, Inha University, Incheon, Republic of Korea; ^2^Research Center for Controlling Intercellular Communication, College of Medicine, Inha University, Incheon, Republic of Korea; ^3^Program in Biomedical Science and Engineering, Inha University, Incheon, Republic of Korea; ^4^Department of Kinesiology, Inha University, Incheon, Republic of Korea; ^5^Department of Urology, College of Medicine, Inha University, Incheon, Republic of Korea

**Keywords:** beta-hydroxybutyrate, fat browning, mitochondrial biogenesis, adipokine, exercise, ketogenic diet

## Abstract

**Introduction:**

Despite evidence suggesting that metabolic intermediates like β-HB influence white adipose tissue (WAT) metabolism, the precise molecular mechanisms remain unclear. The aim of this study was to investigate the impact of beta-hydroxybutyrate (β-HB) on the fat browning program and to explore the underlying molecular mechanisms using both *in vitro* and *in vivo* models. We assessed the effects of β-HB on fat browning in adipocytes using 3T3-L1 cells and rat models.

**Methods:**

We evaluated the effects of β-HB on fat browning, thermogenesis, lipid accumulation, adipokine expression, and mitochondrial biogenesis by treating mature 3T3-L1 adipocytes with sodium β-HB for 24 h or by continuously exposing preadipocytes to β-HB during the 8-day differentiation process. Male Sprague Dawley rats were divided into control, exercise only (EX), ketogenic diet only (KD), and combined exercise and ketogenic diet (KE) groups for an 8-week intervention involving diet and/or exercise. After intervention, we evaluated WAT histology, plasma lipids and adipokines, and the expression of markers related to fat browning, thermogenesis and mitochondrial biogenesis in WAT of rats.

**Results:**

In our adipocyte culture experiments, β-HB reduced intracellular lipid accumulation by enhancing lipolysis and stimulated the expression of thermogenic and fat browning genes like uncoupling protein 1 (UCP1), PR domain containing 16 (PRDM16), and adipokines such as fibroblast growth factor 21 (FGF21) and Fibronectin type III domain-containing protein 5 (FDNC5). Additionally, β-HB activated the AMPK-SIRT1-PGC-1α pathway, with UCP1 and PRDM16 upregulation mediated by β-HB intracellular action and SIRT1 activity. In animal experiments, KE group raised β-HB levels, decreasing body weight and blood lipids. KD with EX promoted WAT browning possibly via AMPK-SIRT1-PGC-1α, augmenting PRDM16, UCP1, FGF21, and FNDC5 expression.

**Conclusion:**

β-HB induction via KD and/or EX shows potential in promoting WAT browning by activating mitochondrial biogenesis, lipolysis, and thermogenesis, suggesting that dietary and physical intervention inducing β-HB may benefit metabolic health.

## 1 Introduction

During fasting, energy balance is maintained by breaking down triglyceride (TG) in white adipose tissue (WAT) into fatty acids, producing ketone bodies (β-hydroxybutyrate, acetoacetate and acetone). Among these, β-HB stands out as the most abundant ketone body synthesized predominantly during glucose shortage, formed in the liver from β-oxidation of fatty acids that are produced via lipolysis of adipose tissues (1). It serves as a crucial indicator of energy metabolism, utilized as a significant energy source in the brain, skeletal muscle, heart and other peripheral tissues. However, the contributions of brown adipose tissue (BAT) and browning of WAT depots (beige fat) in energy metabolism under energy-deficient conditions remain incompletely understood, despite their potential importance in regulating energy stores. WAT and BAT are functionally distinct. WAT primarily engages in energy storage and is central to metabolic diseases associated with obesity. In contrast, BAT, rich in mitochondria and containing uncoupling protein 1 (UCP1), is responsible for heat production that positively affects obesity and metabolic diseases. Under certain conditions, WAT can convert to beige fat by mitochondrial biogenesis and upregulation of UCP1, which has similar functions to brown fat (2). This process is referred to as fat browning, and it is considered a promising approach to increasing energy expenditure and improving metabolic health. Converting energy-storing WAT to thermogenic beige fat is considered a promising approach for sustained weight management and metabolic health (3, 4). Therefore, methods to induce fat browning, such as pharmacological and lifestyle interventions, are being explored to combat obesity and metabolic disorders (5). However, challenges and difficulties in developing effective BAT-activating thermogenic enhancers (e.g., sympathomimetics) have prompted interest in non-pharmacological intervention like exercise and diet to enhance fat browning (6).

Beige fat and BAT, rich in mitochondria with UCP1, facilitate non-shivering thermogenesis (NST), supported by an increase in BAT-associated genes, including peroxisome proliferator-activated receptor gamma coactivator 1 alpha (PGC-1α) and PR/SET domain 16 (PRDM16). Additionally, fat browning can be induced by adipomyokines such as irisin, produced by skeletal muscle and/or adipose tissue, which can transform subcutaneous fat to a beige phenotype (7–10). Adipomyokines are dynamically regulated by cold-mimicking adrenergic agonists and non-pharmacological intervention, including exercise and diet. Induces the browning of subcutaneous fat (7). Similarly, fibroblast growth factor 21 (FGF21), another adipomyokine produced by the liver, adipose tissue, and skeletal muscle, is an endocrine activator that increases UCP1 expression in both WAT and BAT in rodents and human (11–13). Energy expenditure by cold-induced shivering thermogenesis or NST are two major mechanisms regulating body temperature and energy homeostasis, mediated by the sympatho-thyroid-adrenal axis (14) or BAT activation (15), respectively. Among adipomyokines, irisin and FGF21 are suggested to be jointly secreted in response to cold exposure, contributing to thermogenesis and energy expenditure (12).

In our previous study, we demonstrated that acute elevation of lactate and β-hydroxybutyrate (β-HB) levels induced by single bout of high-intensity exercise in a fasted state can regulate whole-body metabolism and adipomyokine secretion (16). β-HB, the most abundant ketone body, is an essential energy carrier produced from fatty acids during glucose depletion (1, 17). Interestingly, both β-HB and lactate, metabolic intermediates produced during fasting or exercise, have been shown to promote fat browning through redox-dependent adaptations in murine WAT (18). However, the specific cellular signaling pathways and adipomyokine expression in WAT induced by physiological levels of β-HB resulting from chronic diet and/or exercise interventions are not yet fully understood, despite the potential roles of β-HB in fat browning and NST.

The objective of this study is to investigate the roles of β-HB in regulating the fat browning program and adipomyokine expression *in vitro* and WAT in rats on a low-carbohydrate/high-fat ketogenic diet (KD) and/or undergoing aerobic exercise training. By understanding the underlying mechanisms of β-HB-induced fat browning and its potential as a non-pharmacological intervention, important insights can be gained into the development of effective therapeutic strategies against metabolic diseases.

## 2 Materials and methods

### 2.1 Cell culture

The 3T3L1 pre-adipocytes (ATCC; CL-173, Manassas, VA, United States) were cultured and maintained in Dulbecco’s Modified Eagle’s Medium (DMEM; Gibco-BRL, Grand Island, NY, United States) supplemented with 10% heat-inactivated newborn calf serum (NBCS; Gibco-BRL), 100 U/mL penicillin, and 100 μg/mL streptomycin at 37°C in a humidified atmosphere (95% air and 5% CO2) incubator. Two days after the cells become confluent, we replaced the proliferation medium with MDI induction medium, which consisted of DMEM containing 10% fetal bovine serum (FBS; Gibco-BRL), 1 μM dexamethasone (D2915, Sigma-Aldrich, St. Louis, MO, United States), 0.5 mM 3-isobutyl-1-methylxanthine (IBMX; 17018, Sigma-Aldrich) and 10 μg/mL insulin (16634, Sigma-Aldrich). The cells were then cultured for an additional 48 h before replacing the medium with differentiation medium (DM), which consisted of DMEM supplemented with insulin (10 μg/mL) every 2 days. On day 8 or during differentiation, we treated cells with sodium β-HB (Sigma-Aldrich) for the indicated time in DMEM with low glucose (1 g/L).

### 2.2 Analysis of cell viability

The viability of 3T3L1 was measured using a 3-(4,5-dimethylthiazol-2-yl)-2,5-diphenyltetrazolium (MTT) assay, as described previously (19), to determine the cytotoxicity of β-HB. Briefly, MTT solution (0.5 mg/mL) was added to each well, followed by incubation at 37°C for 2 h. After removal the supernatant, formazan crystals were lysed with dimethyl sulfoxide at 37°C for 30 min. The optical density was measured using a microplate reader at 570 nm.

### 2.3 Oil red O staining

Staining was conducted upon 3 T3-L1 pre-adipocytes either during the differentiation process or upon completion of differentiation, after treatment with 4 mM sodium β-HB. Adipocytes were fixed with 3.7% formaldehyde (Sigma-Aldrich, MO, United States) in phosphate-buffered saline (PBS) for 10 min at room temperature (RT), followed by washing twice with PBS. Then, a filtered mixture of Oil-Red O solution in isopropanol and water at a ratio of 3:2 was layered onto cells for 30 min. After a 1-min incubation, the cells were washed with PBS three times, and the stained lipid droplets were captured using a microscope (MC170-HD, Leica Biosystems, Wetzlar, Germany). To quantify the intracellular lipid droplets, the stained cells were dissolved in isopropanol (Sigma-Aldrich) and their optical intensities were measured using a microplate reader at 570 nm.

### 2.4 Animal experiment design

To increase circulating levels of β-HB, we treated rats with aerobic exercise and/or KD. Eight-week-old (230–250 g) male Sprague Dawley (SD) rats that were maintained at temperature ranged 22–25°C, 40–60% of humidity, with a 12 h light/dark cycle were randomly divided into four groups (*n* = 8 per group): sedentary control (CON), control with moderate-intensity exercise (EX), a sedentary group with KD (KD), and a group with moderate intensity exercise and KD (KE). Following previous studies that reported KD-induced increases of ketone bodies in blood (20, 21), the KE and KD groups were fed with KD [modified AIN-76A (7.24 kcal/g) with 3.2% carbohydrates, 8.6% protein, 75.1% fat, approximately 13% water, fiber, and ash; Bio-Serv F3666, Frenchtown, NJ] while CON group was fed with chow diet [D10001 diet (3.90 kcal/g) with 67.7% carbohydrate, 11.5% fat, 20.8% protein; Research Diets Inc., Framingham, MA] for 8-weeks with or without exercise. We measured the levels of β-HB in spot blood before exercise (Pre-exercise baseline; Pre), immediately after exercise (Post-exercise; Post) and 1 h after exercise recovery time (RE) on the final day of exercise intervention. To exclude the acute effects of exercise on whole body metabolism, we sacrificed the rat for collecting adipose tissue and blood 3 days after 8-week exercise intervention, under anesthesia with intraperitoneal injection of Zoletil (50 mg·kg^−1^) and intramuscular Rompun (5 ~ 10 mg·kg^−1^).

### 2.5 Ethics statement

The care of animals was conducted according to the Guide for Care and Use of Laboratory Animals of the National Institutes of Health of Korea. The study protocol was approved by the Institutional Animal Care and Use Committee (IACUC) of Inha University (INHA 181004–597, Incheon, Korea).

### 2.6 Exercise protocol

The rats in the EX and KE groups underwent aerobic exercise training for 8 weeks on a motorized, speed-controlled treadmill apparatus (DJ2-242, Dual Treadmill Daejong, Ltd., Korea) for 40 min under dim lighting conditions during the daytime, with a 0° inclination. After 1 week of acclimation, EX and KE groups were adapted to the treadmill exercise without inclination at 5–10 m/min (gradually increasing speed, 20 min/day and 5 days/week) for 1 week. After adaptation, the animals underwent aerobic exercise training for 8 weeks. The exercise intensity was gradually increased over the study period. In the initial 4 weeks, the animals performed a 5-min warm-up at a speed of 8 m/min, followed by 22 min at a speed of 15 m/min, and 3-min cool-down (5 m/min) 5 times per week. In the subsequent 4 weeks, the warm-up remained at 5 min at 8 m/min, but the main exercise was increased to 32 min at a speed of 20 m/min, and 3-min cool-down (5 m/min) also 5 times per week.

The animals rested for 3 days after 8-week exercise intervention to exclude the carry-over confounding effects of the last-day exercise on the biological effects of the chronic exercise training. The intensity of exercise was moderate with VO2max of 60–75%, following a previous study (22).

### 2.7 Real-time RT-quantitative PCR

Total RNA was isolated using Trizol reagent (Invitrogen, Carlsbad, CA, United States). RNA (1 μg) was converted to cDNA using a cDNA synthesis kit (Takara Bio Inc., Shiga, Japan) following the manufacturer’s instruction. mRNA expression was measured by quantitative reverse transcriptase polymerase chain reaction (qRT-PCR) using a thermocycler (Bio-Rad, Hercules, CA, United States) and Power SYBR green (Bio-Rad). The relative gene expression was determined using the 2^–ΔΔCT^ method, and Ct values were normalized to the level of GAPDH. The oligonucleotide primers used for amplification are listed in Supplementary Table S1.

### 2.8 Western blot analysis

Protein from WATs or whole cell lysates was prepared using radioimmunoprecipitation assay (RIPA) buffer (50 mM Tris, 150 mM sodium chloride and 1.0% NP-40) containing both protease inhibitors and phosphatase inhibitors cocktail (Sigma–Aldrich). Whole-cell lysates and collected WATs in RIPA buffer were sonicated or homogenized using a Teflon homogenizer, respectively. Aliquots of protein extracts (10–30 μg) were subjected to western blot analysis with appropriate antibody. SDS-PAGE were conducted using 8–15% polyacrylamide and transferred to a nitrocellulose membrane (Pall Corporation, Washington D.C, NY, United States) in transfer buffer (25 mM Tris–HCl, 192 mM glycine and 20% methanol). After 2 h of blocking the membrane with 5% (w/v) fat-free milk in Tris-buffered saline, the membranes were incubated at 4°C for 16 h with primary antibodies (Supplementary Table S2) in 5% (w/v) bovine serum albumin (BSA; Sigma Aldrich). After incubating the membrane with horseradish peroxidase conjugated goat anti-rabbit IgG or goat anti-mouse IgG antibodies at RT, proteins were detected with enhanced chemiluminescence detection kits (ECL; Thermo Fisher Scientific, Waltham, MA, United States) and analyzed using a Chemi doc System (Bio-Rad).

### 2.9 Immunofluorescence analysis

The immunofluorescence staining was conducted to investigate the expression of PRDM16 and UCP1 in 3T3L1. Cells were seeded at a density of 3 × 10^5^ cells on to glass coverslips on a 6-well plate and differentiated on glass coverslips for 10 days. Cells were fixed with 4% paraformaldehyde for 10 min at RT and washed 3 times with PBS. Then, cells were permeabilized with 0.2% (v/v) Triton-X100 for 10 min and blocked with blocking solution (0.5% BSA in PBS) for 1 h, followed by overnight treatment with diluted (1:200) anti-PRDM16 or anti-UCP1 antibody in 5% (w/v) BSA solution at 4°C. Afterward, cells were rinsed 3 times with PBS-T for 5 min and treated with anti-rabbit IgG (H + L) F(ab’) fragment Alexa Fluor 488 conjugate (1:500) diluted in 5% (w/v) BSA for 1 h. Cells were washed 3 times again with PBS-T, the nuclei were stained, and mounted with 4′,6-Diamidine-2′-phenylindole dihydrochloride (DAPI, Invitrogen; P36941) for 24 h. Images of the same field were recorded separately and merged by the fluorescence microscopy (IX83, Olympus, Tokyo, Japan).

### 2.10 Blood analysis

To identify change in blood concentrations of β-HB at Pre, Post, and RE, capillary blood samples were drawn from the rat’s tail on the day of final exercise and β-HB level in the spot blood was quantified using the β-HB meter (FreeStyle Optium Neo H; Abbott, Ill, United States). In addition, β-HB levels at 3 days after intervention in plasma obtained from cardiac blood were quantified using colorimetric β-HB assay kit (Cayman Chemical, Ann Arbor, MI, United States).

Levels of plasma triglyceride (TG), high-density lipoprotein (HDL), low-density lipoprotein (LDL), and total cholesterol (TC) were determined using enzyme-linked immunosorbent assay (ELISA) kit (Labtest^®^, São Paulo, Brazil) following the manufacturer’s instructions. Plasma leptin were measured with an ELISA using commercially available kits (R&D Systems, Minneapolis, MN, United States).

### 2.11 Measurement of adipokines using the xMAP-Luminex multiplex immunoassay

Levels of plasma adipomyokines were measured by the xMAP-Luminex multiplex immunoassay platform (Luminex Co., Austin, TX, United States) with the Multiplex Magnetic Bead panel kit (Millipore, Billerica, MA, United States) following the manufacturer’s instructions. Briefly, magnetic beads with capturing antibody were added to each well of flat-bottom 96 well plate followed by washing twice using a handheld Magnetic Separator Block (Merck Millipore). After adding samples, the plate was wrapped with aluminum foil and incubated with agitation on a plate shaker overnight at 4°C. The well was then washed three times, 25 μL of detection antibodies were placed in each well, and incubated with agitation for 2 h at RT. To detect the chemiluminescence signal, we added streptavidin-phycoerythrin, reacted with agitation for 1 h at RT, and wells were washed three times before adding sheath fluid. Finally, we ran the plate on the Luminex®200™ and the xPONENT software to measure the concentration of adipomyokines simultaneously.

### 2.12 Hematoxylin and eosin staining

To investigate the adipose tissue morphology, WATs were fixed overnight with 4% paraformaldehyde (Sigma-Aldrich) at RT. Paraffin-embedded eWAT was prepared and cut with 5 μm of thickness. Paraffin sections of fat tissues were stained with hematoxylin for 5 min, rinsed with running tap water, and stained with eosin solution for 2 min. The slides were then rinsed with absolute alcohol and mounted using synthetic resin and dried overnight at RT.

### 2.13 Immunohistochemistry

For immunohistochemical staining of UCP1 and PRDM16, adipose tissue slides were deparaffinized in xylene, hydrated in 100 and 70% ethanol, and rinsed in water before heat-mediated antigen retrieval in 10 mM sodium citrate buffer (pH 6.0) for 20 min at 97°C. Slides were blocked with 2.5% horse serum (Gibco-BRL), followed by incubation with rabbit polyclonal UCP1 and PRDM16 primary antibody diluted 1:100 overnight at 4°C. After incubation the slides were washed in PBS for 5 min and then incubated with secondary antibody for 10 min. After washing the slides in PBS, slides were incubated with substrate solution for 1 min and then counterstained with Harris hematoxylin.

### 2.14 Statistical analysis

All statistical analyses in this investigation were conducted using the SPSS v.26 software or Prism ver. 9 (GraphPad Software, San Diego, CA, United States). Data were expressed as the mean ± standard error (S.E.). For the *in vitro* study, differences between groups were analyzed using appropriate tests such as one-sample *t*-test, unpaired *t*-test or Mann–Whitney *U* test for two group comparison, or one-way ANOVA with multiple comparison. In the animal study, continuous variables were tested for normality using the Shapiro–Wilk test. Unpaired *t*-test or Mann–Whitney *U* test were employed to assess group differences for comparing two groups, and one-way ANOVA or the Kruskal–Wallis test were used for comparing three or more groups, based on standard statistical assumptions. After finding significant differences in univariate tests, the Tukey HSD and Mann–Whitney *U* test were used as a *post hoc* analysis to determine the location of significant differences. Pearson’s correlation analysis was applied to assess the correlation between the levels of β-HB and blood lipids.

Differences in groups (CON, EX, KD, and KE) over time (Pre, Post, and 1 h Re) for β-HB were identified using a two-way (4 groups × 3 time points) repeated measures ANOVA. After finding significant differences in multivariate and univariate tests, the Bonferroni test was used as a *post hoc* analysis to determine a location of significant differences. Outlier data was identified by Grubbs’ method and removed. For all statistical tests, significance was set at an alpha level of 0.05.

## 3 Results

### 3.1 β-HB inhibits intracellular lipid accumulation in adipocytes

**Figure 1:**
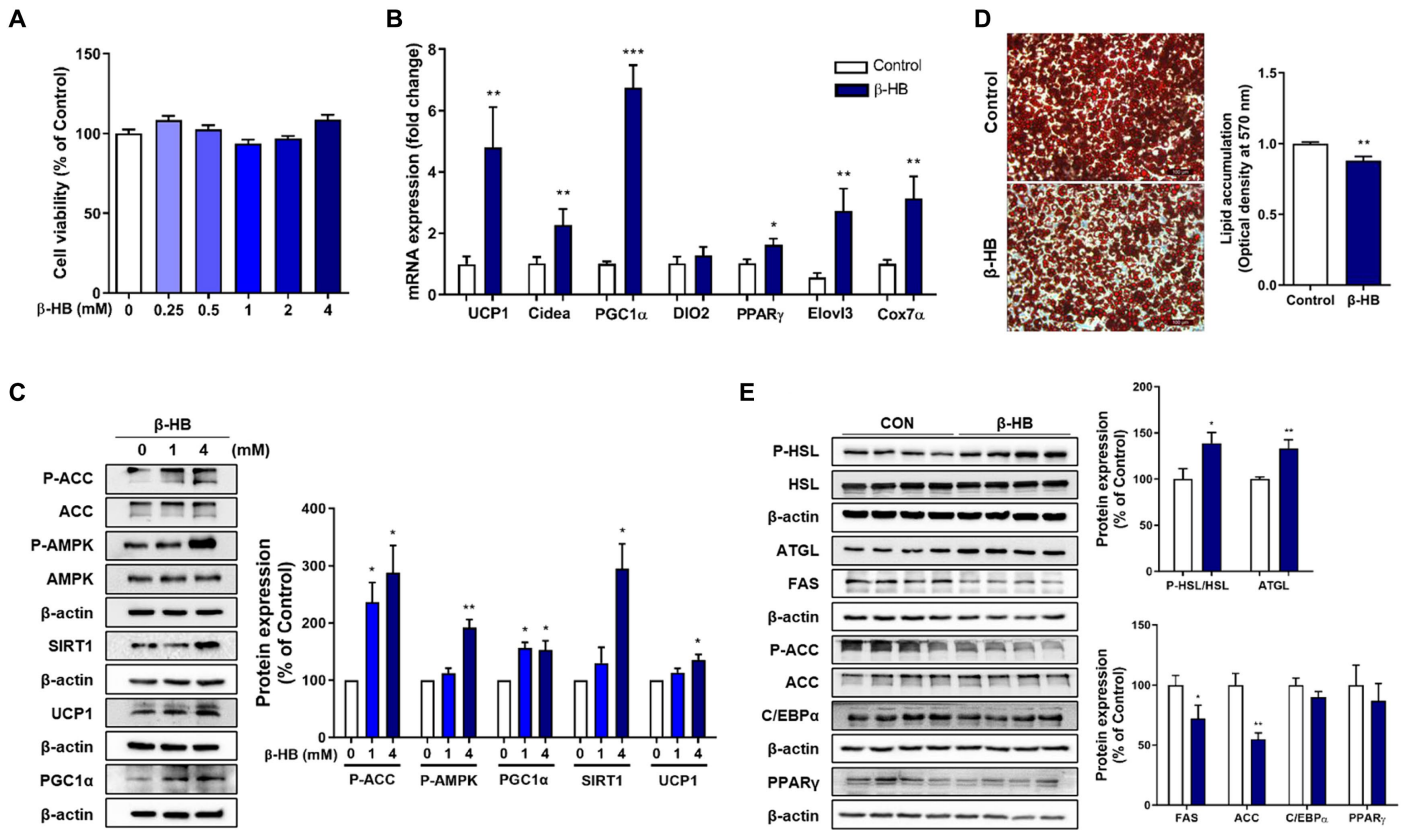
β-HB increases fat browning markers and inhibits lipid accumulation of 3T3-L1 adipocytes. **(A)** Differentiated 3T3-L1 adipocytes treated with β-HB (~4 mM) for 24 h (*n* = 8) showed no significant cytotoxicity. **(B)** mRNA expression levels of thermogenic genes and mitochondrial biogenesis marker genes in differentiated adipocytes treated with vehicle control or β-HB (4 mM, *n* = 7). **(C)** Western blots in differentiated adipocytes treated vehicle or β-HB (4 mM, *n* = 4). **(D)** Quantification of intracellular lipid accumulation in adipocytes treated with β-HB (4 mM) during adipocyte differentiation by optical density of TG extract following Oil-red O staining (*n* = 4). **(E)** Western blot analysis of lipolytic, lipogenic and adipogenic proteins in adipocytes treated with β-HB (4 mM) during differentiation (*n* = 4). *p < 0.05, **p < 0.01, ***p < 0.001 compared with vehicle-treated control using **(C)** one sample *t*-test or **(A,B,D,E)** Mann–Whitney *U* test. All data are shown as the means ± SEM. HSL, hormone-sensitive lipase; ATGL, adipose triglyceride lipase; FAS, fatty acid synthase; AMPK, AMP-activated protein kinase; ACC, acetyl-CoA carboxylase; C/EBPα, CCAAT/enhancer-binding protein alpha; PGC-1α, PPAR-gamma coactivator 1 alpha; PPAR-γ, peroxisome proliferator-activated receptor-gamma, SIRT1, sirtuin 1.

When we treated the mature 3T3-L1 adipocytes with β-HB (~4 mM), no cytotoxicity was observed (Figure 1A). In β-HB treated differentiated adipocytes, mRNA levels of thermogenic genes including *UCP1*, cell death including DFFA like effector A (*Cidea*), and elongation of very long chain fatty acids protein 3 (*Elovl3*), a master regulator of mitochondrial biogenesis (*PGC-1α*) and a mitochondrial marker (cytochrome C oxidase-7*α* 1; *Cox7a1*) were significantly higher than those in vehicle-treated controls (Figure 1B). Consistent with upregulation of mRNA expression for mitochondrial biogenesis and thermogenesis, β-HB upregulated SIRT1, PGC-1α and UCP1 protein expression (Figure 1C). In addition, we observed that β-HB significantly increased the level of p-AMPK and inhibitory phosphorylation of its downstream protein, ACC. Since we observed that β-HB upregulated the mitochondrial biogenic (SIRT1-PGC-1α) and thermogenic factors (UCP1) and anti-lipogenic pathway (p-AMPK/p-ACC), we evaluated the effects of β-HB on adipocyte differentiation and lipid accumulation. When we treated 3T3-L1 pre-adipocytes with differentiation media containing β-HB (4 mM) every other day for 8 days, β-HB-treated adipocytes showed significantly low intracellular lipid accumulation as compared to vehicle-treated controls (Figure 1D). Translational levels of C/EBPα and PPAR-γ, two master regulators of adipogenesis were not significantly different, instead, the levels of acetyl-CoA carboxylase (ACC) and fatty acid synthase (FAS), primary *de novo* lipogenic enzymes converting acetyl-CoA to malonyl-CoA and subsequent fatty acid synthesis, were significantly downregulated by β-HB. In contrary, the levels of key lipolysis-related proteins such as adipose triglyceride lipase (ATGL) and hormone-sensitive lipase (HSL) were significantly elevated by β-HB (Figure 1E). The effects of β-HB at physiologically relevant concentration (1 ~ 2 mM) on the lipolytic and anti-lipogenic enzymes in adipocytes were consistent (Supplementary Figure S1).

### 3.2 Upregulation of thermogenic and fat browning gene expression by β-HB during adipocyte differentiation

**Figure 2:**
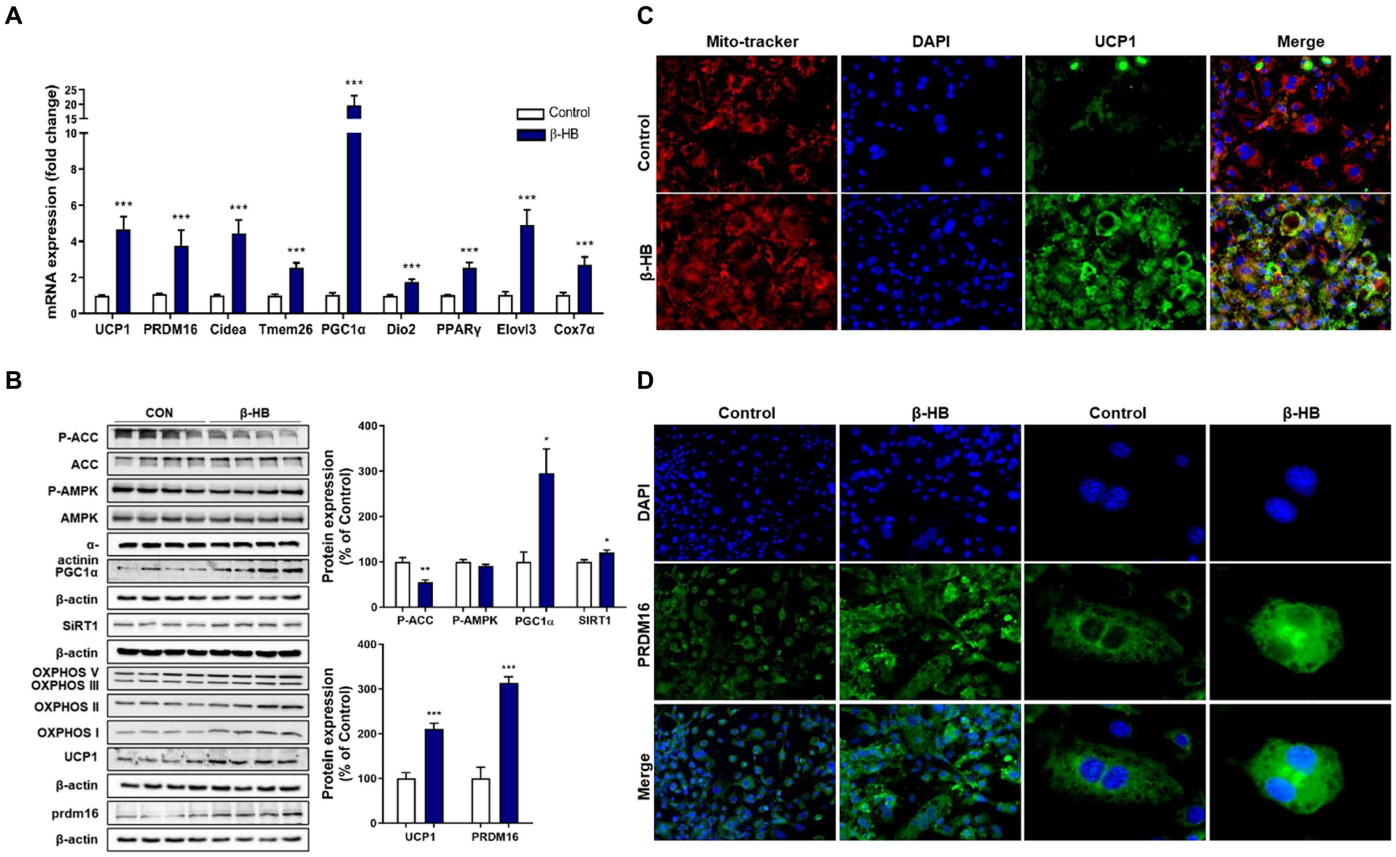
β-HB activates fat browning factors during the differentiation of 3T3-L1 adipocytes. **(A)** mRNA expression levels of thermogenic and fat browning-related genes in adipocytes treated with β-HB (4 mM) during the differentiation (*n* = 7). **(B)** Western blot analysis of thermogenic and fat browning-related proteins in adipocyte treated with β-HB during the differentiation (*n* = 4). **(C,D)** Representative immunofluorescence findings of **(C)** UCP1, mitochondria and **(D)** PRDM16 in adipocytes that were treated with vehicle control or β-HB during adipocyte differentiation. *p < 0.05, **p < 0.01, ***p < 0.001 compared with vehicle-treated control using the Mann–Whitney *U* test. All data are shown as the means ± SEM. Cidea, cell death inducing DFFA like effector A; PGC-1α, PPAR co-activator-1α; COX7α, Cyto C oxidase-7α; PRDM16, PR/SET domain 16; DIO2, iodothyronine deiodinase 2; Tmem26, transmembrane protein 26; and Elovl3, elongation of very long chain fatty acids protein 3.

β-HB-induced inhibition of lipid accumulation without suppression of adipogenic regulators (i.e., C/EBPα and PPAR-γ) may be caused by increased abundancy of mitochondria and mitochondrial fatty acid oxidation, a characteristic of brown adipocyte. Therefore, we next evaluated whether β-HB induce fat browning genes during the differentiation of 3T3-L1 cells. β-HB significantly increased the mRNA levels of thermogenic genes including *UCP1*, *Cidea*, *Dio2* and *Elovl3*, markers of beige adipocytes including *PRDM16* and *Tmem26*, a master regulator of mitochondrial biogenesis (*PGC-1α*) and a mitochondrial marker (*Cox7a1*), as well as *PPAR-γ*, a major regulator of both white and brown adipogenesis (Figure 2A).

The AMPK plays not only pivotal roles in fatty acid oxidation, mitochondrial biogenesis, regulation of thermogenic genes, but also inhibits adipogenesis via suppression of PPAR-γ/C/EBPα pathway (23, 24). β-HB could not significantly alter the p-AMPK level when we treated β-HB during adipocyte differentiation. SIRT1 can directly deacetylate and activate PGC-1α in hepatocytes (25), which may share mechanism to promote mitochondrial biogenesis and browning of WAT (26, 27). We observed that β-HB significantly increased the expression of PGC-1α and SIRT1. Concurrently, we observed the up-regulation of fat browning-related proteins, including UCP1 and PRDM16, and the expression of mitochondrial OXPHOS complex (Figure 2B). These effects were also observed in cells treated with low concentration (1 ~ 2 mM) of β-HB (Supplementary Figure S2). In addition, in β-HB treated cells, the increased mitochondrial abundancy were observed (Figure 2C). Consistently, we observed the upregulation of UCP1 in β-HB-treated cells by addressing the intracellular localization of these proteins in the analysis of fluorescent immunocytochemistry, which was co-localized with the abundancy of mitochondria (Figure 2C). Furthermore, β-HB treatment increased the expression and nuclear translocation of PRDM16 (Figure 2D). Therefore, these results indicate that β-HB upregulates thermogenic and mitochondrial biogenic factors during adipocyte differentiation without inhibition of adipogenesis.

### 3.3 Effects of β-HB on lipid accumulation in mature adipocytes

**Figure 3:**
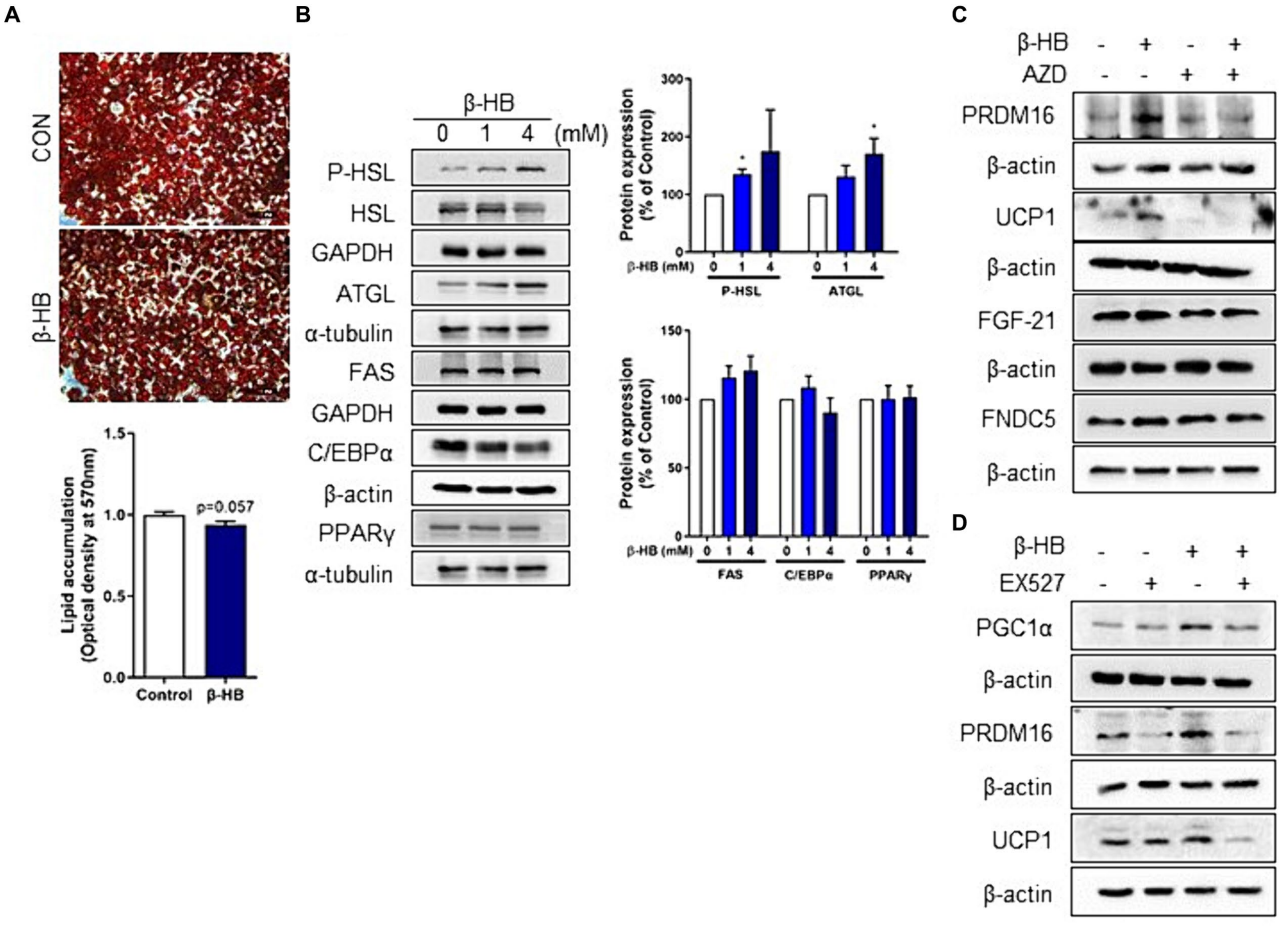
Intracellular β-HB activates lipolysis and induces browning of mature adipocytes in a SIRT1-dependent manner. **(A)** Quantification of intracellular triglyceride in differentiated adipocytes using Oil-red O staining. **(B)** Western blot analysis of lipolytic, lipogenic and adipogenic proteins in differentiated adipocytes treated with β-HB (4 mM, *n* = 4). **(C)** Western blot analysis of UCP1, PRDM16 and adipokines by β-HB (4 mM) in differentiated adipocytes that were treated with monocarboxylate transporter 1 inhibitor (AZD3965, 1 μM) and β-HB (4 mM). **(D)** Western blot analysis of fat browning-related proteins in differentiated adipocytes treated with SIRT1 inhibitor (EX527, 5 μM) and β-HB (4 mM). *p < 0.05compared with vehicle-treated control using Mann–Whitney *U* test. All data are shown as the means ± SEM.

We observed the up-regulation of thermogenic UCP1 expression and pathways associated with mitochondrial biogenesis, i.e., AMPK-SIRT1-PGC-1α in mature adipocytes (Figure 1C). Therefore, we determined the effects of β-HB on the lipid accumulation in mature adipocytes. When we treated 3T3-L1 mature adipocytes with serum-free media containing β-HB (4 mM) for 24 h, we observed a tendency of lipid accumulation decrease in β-HB-treated adipocytes (Figure 3A). Consistently with decreased lipid accumulation by β-HB, the expression of lipolytic proteins (HSL and ATGL), but not of lipogenic protein (FAS), was increased by β-HB (Figure 3B). In addition, the levels of adipogenic proteins (C/EBPα and PPARγ) were not significantly different by β-HB. The effect of β-HB on UCP1, PRDM16, FGF21 and FNDC5 upregulation was abolished by a monocarboxylate transporter 1 (encoded by *SLC16A1* gene) inhibitor, AZD3965, indicating the intracellular action of β-HB in adipocytes (Figure 3C). Next, we evaluated whether upregulation of UCP1 and PRDM16 is dependent on SIRT1 using a SIRT1 inhibitor, EX527. In the presence of EX527, the upregulation of PGC-1α, UCP1 and PRDM16 protein expression by β-HB was abolished (Figure 3D). Combined, these results indicate that activation of fat-browning program by intracellular β-HB may be dependent on the AMPK-SIRT1-PGC-1α pathway.

### 3.4 Regulation of fat browning related adipokine in adipocyte by β-HB

**Figure 4:**
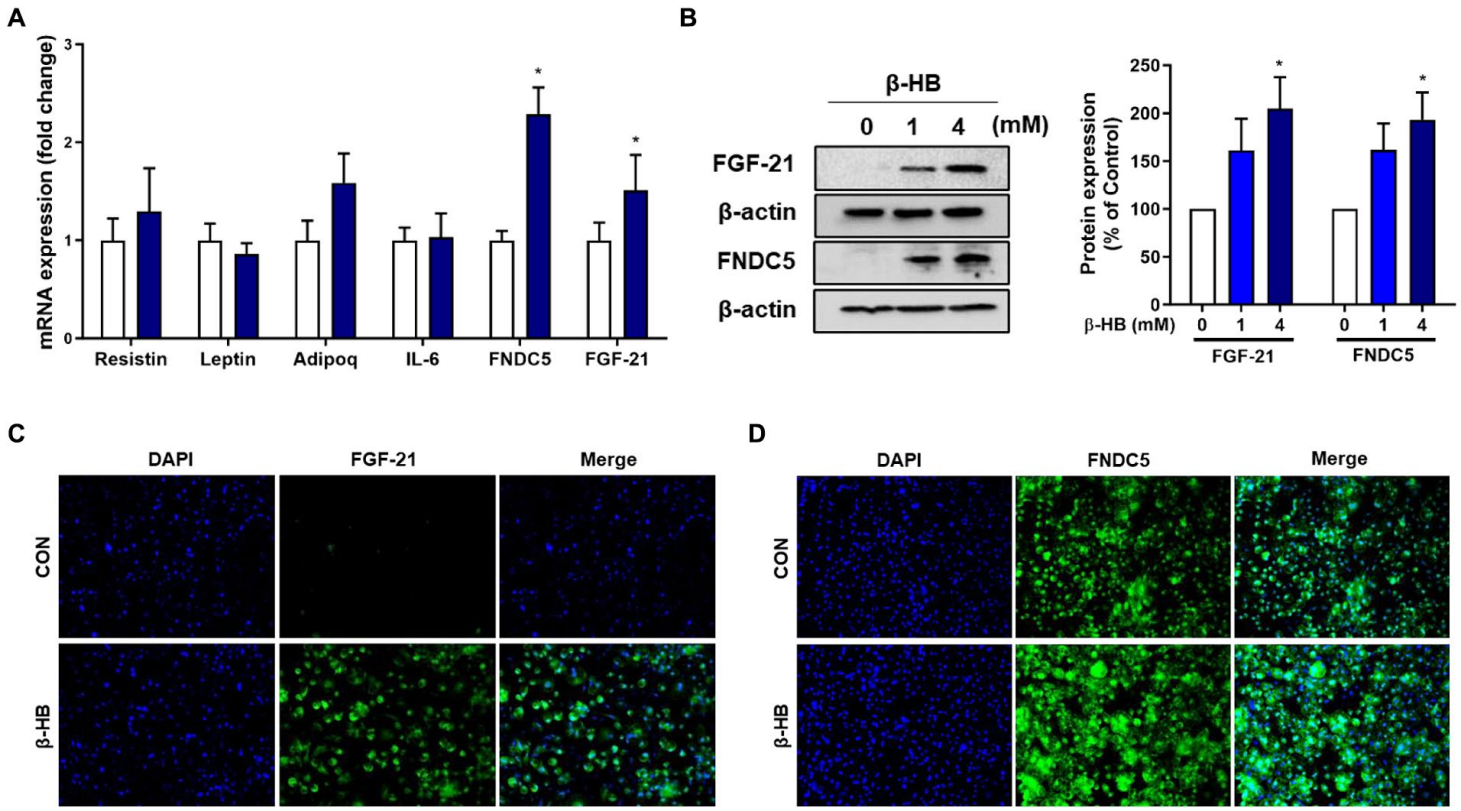
β-HB upregulates FGF21 and FNDC5 expression in 3T3L1 adipocytes. **(A)** mRNA expression levels of adipokines in adipocytes treated with β-HB (4 mM) during adipocyte differentiation (*n* = 4). **(B)** Protein levels of FGF21 and FNDC5 in mature adipocytes treated with β-HB (*n* = 4). **(C,D)** Representative immunofluorescence findings of **(C)** FGF21 and **(D)** FNDC5 in β-HB-treated adipocytes. *p < 0.05 compared with vehicle-treated controls using the Mann–Whitney *U* test. All data are shown as the means ± SEM.

Next, we evaluated whether β-HB induce adipokines, including the irisin precursor, Fibronectin Type III Domain Containing 5 (FNDC5), resistin, leptin, adiponectin and fibroblast growth factor 21 (FGF21) in mature adipocytes. β-HB significantly up-regulated mRNA levels of FNDC5 and FGF21, but not others (Figure 4A and Supplementary Figure S3A). Irisin and FGF21 act as adipokines beiging adipocytes that attenuate diet-induced obesity by stimulating thermogenesis and increase insulin sensitivity (28, 29). Treatment of 3 T3-L1 cells with β-HB significantly upregulated the protein expressions of FNDC5 and FGF21 (Figure 4B and Supplementary Figure S3B). We evaluated further effects of β-HB treatments on the expression of FNDC5 and FGF21 by assessing the localization of these proteins by fluorescent immunocytochemistry. As shown in Figures 4C,D, the β-HB-treated adipocytes highly expressed the intracellular FGF21 comparing with vehicle-treated adipocytes that scanty expressed FGF21. In vehicle-treated controls, some cells express FDNC5, but others express scanty, however, most cells treated with β-HB highly expressed FNDC5.

### 3.5 Effects of ketogenic diet and exercise inducing hyperketonemia on body weight and circulating lipid profile in rats

**Figure 5:**
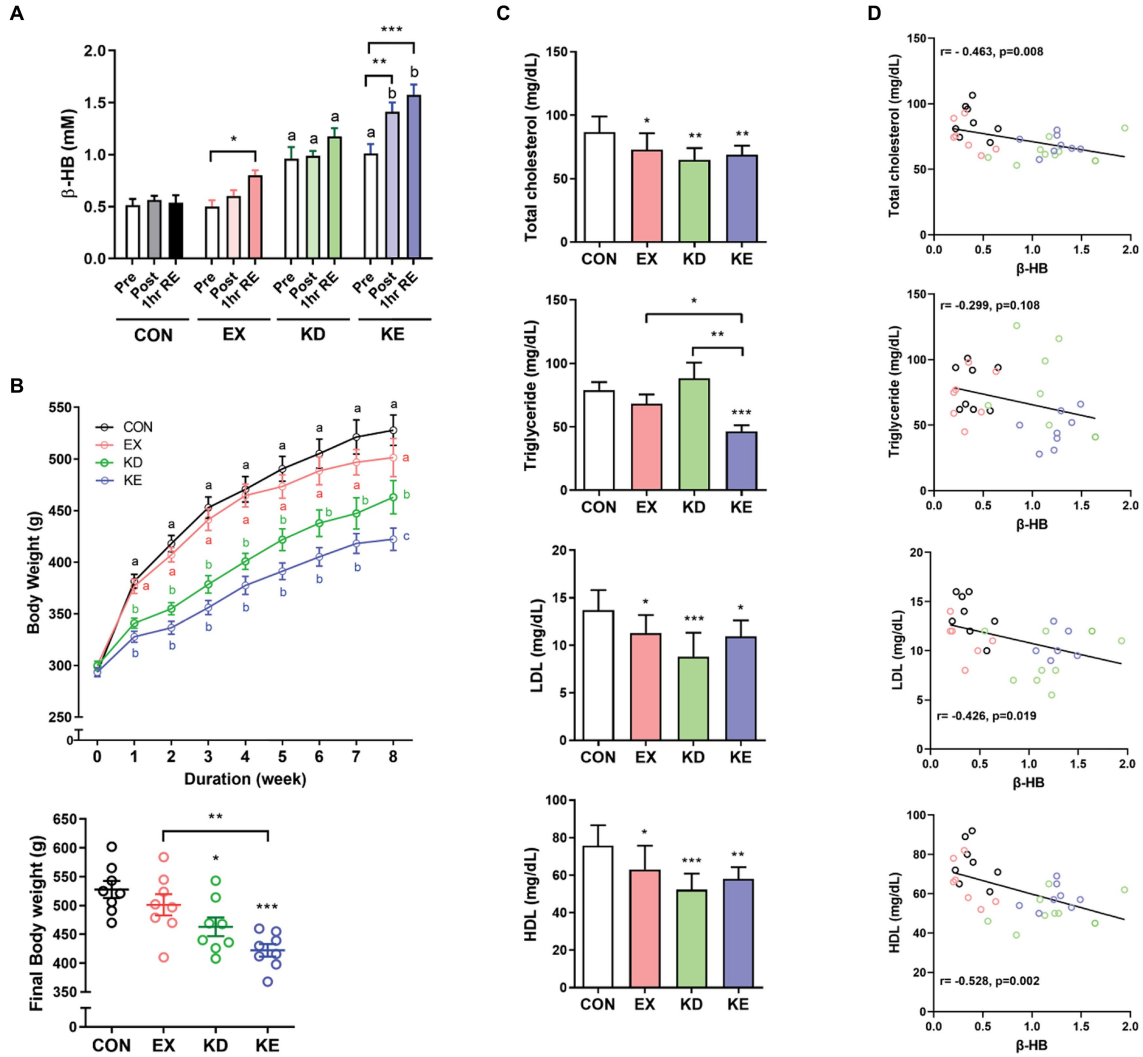
Effects of ketogenic diet (KD) and/or aerobic exercise training (EX) on body weight and blood lipids in rats. **(A)** Plasma β-HB levels were measured before (Pre), immediately after (Post) and 1 h into recovery (1 h RE) after exercise in rats with or KD and/or EX for 8 weeks (*n* = 8, each group). Different characters indicate significant differences compared with control (Pre) using a Two-way (4×3) mixed-measures ANOVA with Tukey’s multiple comparison. *p < 0.05, **p < 0.01 versus the pre of each group by Two-way ANOVA with Dunnett’s multiple comparison. **(B)** The group fed with the ketogenic diet (KD and KE) exhibited distinct weight loss, with the final body weight in the KE group significantly lower than CON and EX groups. Different characters indicate significant difference among group using a Two-way ANOVA with Tukey’s multiple comparison. *p < 0.05, **p < 0.01, ***p < 0.001 by unpaired *t*-test. **(C)** After 8 weeks of diet and/or exercise intervention, levels of plasma lipids at 3 days after intervention to avoid potential carry-over effects of the exercise intervention were measured. TC, total cholesterol; LDL, low-density lipoprotein; HDL, high-density lipoprotein; TG, triglyceride. *p < 0.05, **p < 0.01, ***p < 0.001 by unpaired *t*-test. All data are shown as the means ± SEM. **(D)** Pearson’s correlation analysis showed that increasing levels of β-HB correlated with reduction in blood TC, LDL and HDL, but not with TG. All data are shown as the means ± SEM.

Both exercise (EX) and ketogenic diet (KD) increased the circulating level of β-HB and combined intervention of ketogenic diet and exercise (KE) showed an additive effect. β-HB showed a significant group x time interaction effect (*p* = 0.008) (Figure 5A). Ketogenic diet increased the mean plasma concentration of β-HB (approximately 1 mM in KD and KE at pre-exercise baseline), which was 2-folds higher than the levels in groups fed normal diet (CON and EX). β-HB concentrations immediately after exercise intervention (Post) and at 1 h after recovery period (1 h RE) in KE groups were higher than that in pre-exercise baseline. In EX group, β-HB concentration at 1 h after exercise, but not immediately after exercise (Post) was significantly higher than the concentration at pre-exercise baseline. Mean plasma levels of β-HB at 1 h after exercise in EX and KE groups were 0.8 mM and 1.6 mM, respectively. Regarding body weight, exercise intervention slightly but not significantly reduced body weight, while KD and KE groups showed the significantly lower body weight than CON group. After 8 weeks of intervention, body weight of KE group was significantly lower than both CON and EX group (Figure 5B).

*In vitro* results showed the thermogenic and lipolytic effects of β-HB, therefore we evaluated the effects of exercise and/or diet-induced hyperketonemia on blood lipid levels. We sacrificed the animals 3 days after intervention to avoid potential carry-over effects of the exercise intervention, and collected tissues and cardiac blood. Mean plasma concentration of β-HB in the KD (1.15 ± 0.40 mM) and KE (1.23 ± 0.19 mM) groups was over 3-fold higher than that in the CON (0.39 ± 0.15 mM) or EX group (0.34 ± 0.16 mM). TC, LDL and HDL levels in EX, KD and KE groups were significantly lower than those in CON. The TG level in KE group was significantly lower than that in other groups, while the TG level in the EX or KD groups was comparable to that in the CON group (Figure 5C). Interestingly, we observed a significant correlation between the plasma β-HB level and blood lipid levels at the end of 8-week intervention. The β-HB levels negatively correlated with levels of TC (*r* = −0.463, *p* = 0.008), HDL (*r* = −0.528, *p* = 0.002), and LDL (*r* = −0.426, *p* = 0.019), but not with TG (*r* = −0.299, *p* = 0.108) (Figure 5D).

### 3.6 Effects of ketogenic diet and/or exercise intervention on browning of WAT

**Figure 6:**
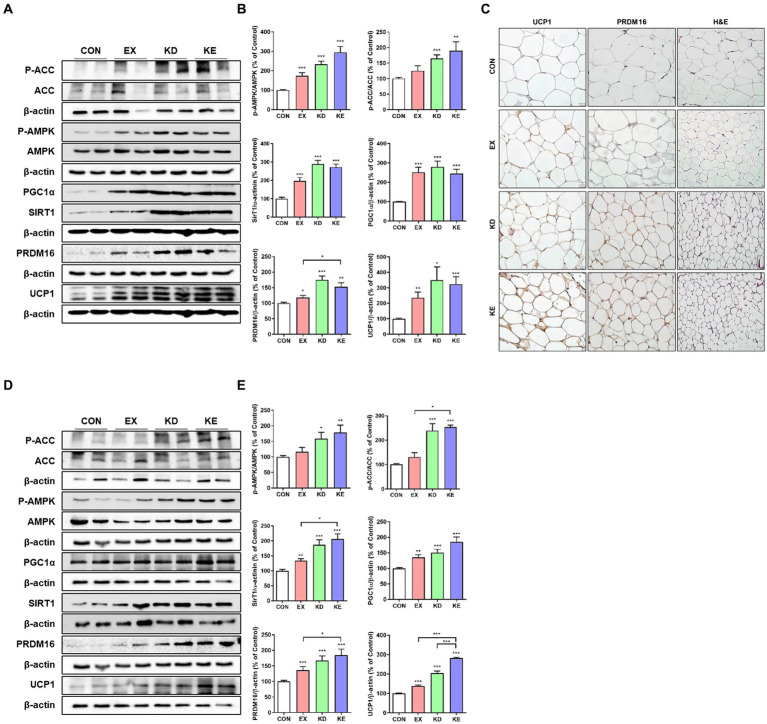
Ketogenic diet and aerobic exercise training activates fat browning program in white adipose tissues of rats. Protein expression of AMPK-SIRT1-PGC-1α pathway and thermogenic (UCP1) and fat browning (PRDM16) marker proteins in **(A,B)** eWAT and **(D,E)** iWAT of rats are shown. Graphs indicate the semiquantitative analysis of the protein levels, and each blot is representative data (*n* = 7–8 in each group). *p < 0.05, **p < 0.01, ***p < 0.001 by the Mann–Whitney *U* test. All data are shown as the means ± SEM. **(C)** Representative immunohistochemical findings of UCP1 and PRDM16 expression in eWAT.

The AMPK plays a critical role in regulating cellular energy mechanism and acts an important role important role in regulating the browning process of WAT in a SIRT1–PGC-1α-dependent manner (30). Therefore, we next evaluated the effects of aerobic exercise with ketogenic diet on pathways related to mitochondrial biogenesis (AMPK-SIRT1-PGC-1α) and fat browning-related proteins (PRDM16 and UCP1) in WAT. As shown in Figures 6A,B, the levels of phosphorylated AMPK (p-AMPK/AMPK) and its downstream target of phosphorylated ACC (p-ACC/ACC) in the KD or KE group were significantly higher than those of CON. EX group showed a higher p-AMPKACC/AMPK level than CON group. Levels of SIRT1 and PGC-1α expression in intervened groups (EX, KD, and KE) were significantly higher than CON. PRDM16 is highly enriched in brown adipocytes and activates a brown fat gene program (31). When PRDM16 expresses in WAT, white adipocyte genes are suppressed while the expression of several proteins involved in browning phenotype including UCP1 and PGC-1α are stimulated (31–33). In our result, the simultaneous upregulation of PRDM16 and UCP1 was observed in EX, KD, and KE groups. In particular, we confirmed a significant increase in PRDM16 levels in the KE group compared to the EX group in eWAT. In immunohistochemical analysis, ketogenic diet fed rats (KD and KE groups) showed the significantly lower size of fat droplet than CON group. EX group also has the slightly lower size of fat droplet. Intensities of UCP1 and PRDM16 staining in KE group were clearly higher than CON (Figure 6C). In subcutaneous iWAT, as shown in Figures 6D,E, the pattern of upregulation in the levels of proteins that are associated with fat browning was consistent with those in eWAT. Specifically, upregulation of AMPK-SIRT1-PGC-1α pathway were observed in iWAT of KD and KE groups, compared with the CON. Furthermore, the levels of PRDM16 and UCP1 in iWAT of the all intervened groups were significantly higher compared with the CON. In particular, we observed significantly higher increases in the levels of PRDM16 and UCP1 in the KE group compared to the EX group. Moreover, the expression of UCP1 was even higher in the KE group compared to the KD group.

### 3.7 Changes in adipokine expression after ketogenic diet with/without aerobic exercise training

**Figure 7:**
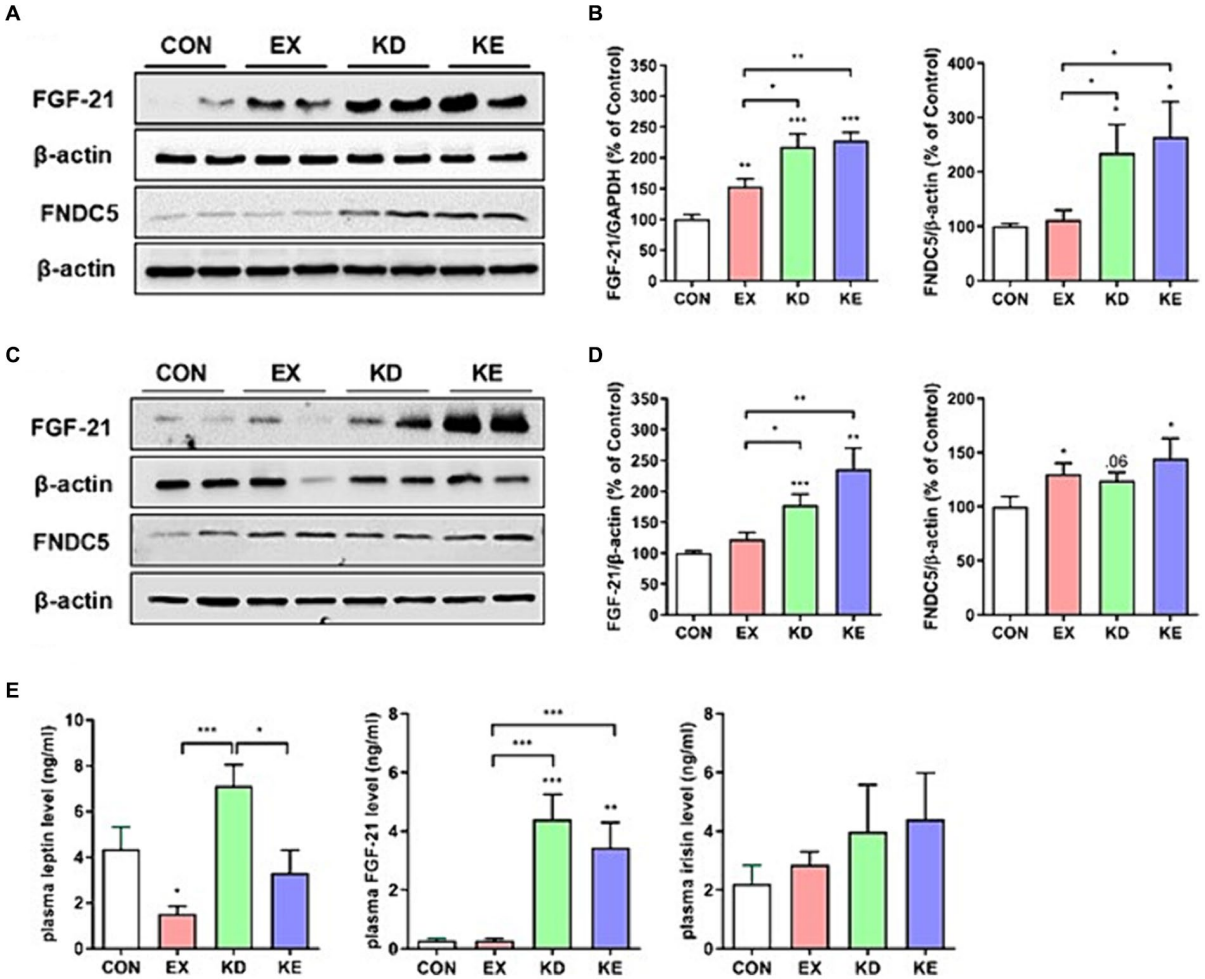
Effects of a ketogenic diet and/or aerobic exercise training on fat browning-related adipokines expression in white adipose tissue and plasma. Protein expression of FGF21 and FNDC5 in **(A,B)** eWAT and **(C,D)** iWAT of rats are shown (*n* = 7–8 in each group). Graphs indicate the semiquantitative analysis of the protein levels, and each blot is representative data. **(E)** Plasma levels of leptin, FGF21 and irisin were presented (*n* = 7–8 in each group). *p < 0.05, **p < 0.01, ***p < 0.001 by the Mann–Whitney *U* test. All data are shown as the means ± SEM.

To confirm the *in vitro* findings of β-HB-induced upregulation of fat browning adipokines (i.e., FGF21 and irisin), we evaluated the effects of exercise with ketogenic diet on the levels of FGF21 and FNDC5 (a precursor of irisin). Given that FGF21 and irisin are adipomyokines involved in fat browning (7, 12, 34) and activate AMPK-SIRT1-PGC-1α pathway (35, 36), we measured the expression levels of FGF21 and FNDC5 in eWAT. The expression levels of FGF21 and FNDC5 protein were significantly higher in KD and KE groups than those in CON and EX groups. In EX group, FGF21, but not FDNC5 protein expression was significantly higher than CON (Figures 7A,B). We also evaluated the expression of FGF21 and FNDC5 in iWAT to confirm the effects of intervention on the levels of these adipomyokines regardless of source of WAT. We found that the expression level of FGF21 in iWAT of KD and KE groups was significantly higher than that of CON. In addition, the level of FNDC5 in iWAT was significantly higher in EX and KE groups than those in CON (Figures 7C,D).We found that the plasma levels of FGF21 in KD and KE groups were significantly higher than CON and EX groups, and the level of FGF21 in EX was comparable with CON. Levels of irisin in plasma of KD and KE were 2-fold higher, but not significant, than the level of CON. Regarding the effects of KD and/or exercise on the levels of leptin, the level of KD group was higher, but EX group was lower than CON group. The level of KE group was comparable to CON, but lower than KD group (Figure 7E).

## 4 Discussion

Our study provides evidence that β-HB can activate the fat browning program, as demonstrated by the up-regulation of thermogenic UCP1 and PRDM16 expression, accompanied by the activation of signaling pathways involved in mitochondrial biogenesis in adipocytes. The *in vitro* evidence was supported by an *in vivo* study involving a KD and/or exercise intervention, which confirmed the activation of fat browning-associated mechanisms, particularly in rats with exercise training and hyperketonemia.

Specifically, our *in vitro* results demonstrated that β-HB activates AMPK-SIRT1-PGC-1α pathway and up-regulates UCP1 expression. PGC-1α, which is activated by both AMPK and SIRT1 through post-translational modification, is a transcriptional regulator of UCP1 (37). Instead of generating ATP, upregulation of UCP1 produces heat, which may activate AMPK to restore intracellular energy. AMPK activation increases intracellular NAD+ and SIRT1 activation (38, 39), resulting in positive feedback accompanied by PGC-1α induction for mitochondrial biogenesis. The up-regulation of fat browning and thermogenic program may result from the intracellular action of β-HB since we observed that the effects were inhibited by a β-HB transporter (MCT1) inhibitor (40). During adipocyte differentiation, β-HB did not inhibit adipogenic signals such as C/EBPα, PPAR-γ, and p-ACC. Instead, β-HB induces marker genes of beige adipocytes, including *PRDM16* and *Tmem26*, as well as UCP1 expression during differentiation. Thus, the inhibitory effects of β-HB on intracellular lipid accumulation during adipocyte differentiation may result from increased lipolysis rather than inhibition of differentiation. While we observed the activation of a fat-browning program *in vitro*, it is unclear whether β-HB itself mediates the up-regulation of PRDM16 and UCP1 *in vivo*. Specifically, we found that the expression levels of UCP1 and PRDM16 protein in both iWAT and eWAT of the KD and KE groups were significantly higher than those in the CON group. Moreover, the UCP1 and PRDM16 levels in the iWAT of the KE group were even higher than those in the EX or KD groups. The post-exercise level of β-HB in the KE group was significantly higher than in other intervention groups. This suggests the possibility of a threshold level of β-HB that up-regulates PRDM16 and UCP1. Another explanation could be that the response to exercise stimuli differs according to the ketogenic diet, or vice versa. Specifically, the response to exercise under hyperketonemic diet conditions, characterized by an abundance of fat-browning gene expression, may be more sensitive than that under control diet conditions. Conversely, the response of the fat browning program to β-HB may be sensitive under condition of combined exercise. In our previous study evaluating the effects of single bout exercise in a fasted state, we observed that the expression of UCP1 and PRDM16 in eWAT of rats was upregulated, which was not the case with fasting only (16). Combined, the exercise intervention in a hyperketonemic state may augment the fat browning effect of β-HB. In addition, a ketogenic diet which may induce hepatic lipid accumulation (41) can be attenuated by exercise intervention. Therefore, in the KD group, hepatic factors can attenuate the effects of β-HB on adipose tissue browning. However, this speculation requires further evaluation using an *in vivo* system to assess the potential risk of ketogenic diet, and to monitor exercise-induced sympathetic activation of fat browning, hepatic metabolism and energy expenditure under a ketogenic diet (42). Our study highlights the need for further vivo studies to elucidate the detailed mechanisms of combined effects of exercise plus hyperketonemia on fat browning. Specifically, the effects of adipose tissue-specific inhibition of β-HB synthesis or MCT1 activity on fat browning induced by hyperketonemia may elucidate the causality of β-HB-induced fat browning resulting from a ketogenic diet and exercise.

Given that adipomyokines like irisin (FNDC5) and FGF21 contribute to fat browning (34), β-HB-induced upregulation of these myokines *in vitro* and *in vivo* may activate fat browning in adipocytes. It has been reported that adipose-derived FGF21 increases the expression of thermogenic genes like UCP1 in thermogenically competent WAT of mice, which is dependent on PGC-1α activation and does not alter adipose differentiation (11). In addition, it has been reported that FGF21 is induced by hyperketonemia intervention in the liver (43). The KE group showed up-regulation of UCP1 and PRDM16 in eWAT, but not the KD group, even though both groups showed hyperketonemia and up-regulation of FGF21 in eWAT and blood. Plasma FGF21 level is up-regulated by a ketogenic diet in human (44); therefore, the up-regulation of FGF21 in hyperketonemia groups (KD and KE) may contribute to the upregulation of UCP1 and PRDM16 in WAT through differential pathways in an exercise stimulation-dependent manner. It has been reported that irisin also upregulates UCP1 and PRDM16 and inhibits adipocyte differentiation in human adipocytes (45). The β-HB-induced activation of AMPK-SIRT1-PGC-1α pathway may be a key mechanism of β-HB inducible fat browning in this study. The expression and activity of PCG-1α is regulated by two metabolic sensors, AMPK (46, 47) and SIRT1 (25), through phosphorylation and deacetylation, respectively. In addition, PGC-1α is a regulator of adipomyokines, including FGF21 and FDNC5. Therefore, the β-HB may contribute to fat browning through the PGC-1α-mediated regulation of genes involved in browning, thermogenesis and adipokine expression.

The activation of membranous G-protein coupled receptor 106A (GPR106A, also known as HCAR2) by β-HB (half maximal effective concentration [EC50] is ~770 μM) inhibits lipolysis (48). Therefore, our *in vitro* results indicate the intracellular actions of β-HB that up-regulate lipolytic enzymes rather than anti-lipolysis by activation of membranous GPR106A receptor in adipocytes. To confirm that the effects of intracellular β-HB are due to its internalization rather than activation of membranous GPR106A receptor on fat browning, further study will be necessary, including *in vivo* experiments involve inhibiting the MCT1 transporter. Although our *in vitro* experiments using an MCT1 inhibitor suggest that β-HB may have intracellular effects, this needs to be confirmed through additional *in vivo* research. By utilizing intracellular free fatty acid, the increased mitochondrial biogenesis and heat generation by β-HB may contribute to decreased body weight and plasma lipids in rats. The significant correlation between plasma levels of β-HB and TC, LDL, and HDL may indicate the contribution of diet-and/or exercise-induced repeated pulsatile up-regulation of β-HB to the regulation of lipid metabolism. The ketogenic diet itself may not be involved in the decreased level of TG, as the TG level in KD group was not different from the CON. However, combined intervention significantly reduced circulating TG level, consistently with previous studies (16, 49).

Our findings contribute to extending our understanding of the roles of β-HB in fat browning. Firstly, we provided both *in vitro* and *in vivo* evidences of the roles of β-HB in promoting the activation of fat browning program and expression of thermogenic adipomyokines, which is consistent with our previous study in a model of single-bout exercise in a fasted state (16). Through the activation of concerted signaling pathways, β-HB may play roles in differentiation of pre-adipocytes to brown-like adipocytes without inhibiting adipogenesis and in beiging of mature adipocytes. Secondly, our study, to our knowledge, firstly provided the effects of combined intervention of ketogenic diet and exercise on fat browning and lipid metabolism in animals with normal weight. It has been reported that combined ketogenic diet and exercise intervention activates fat-lowering effects in animal model of obesity (49). In studies using non-obese animals, the effects of a ketogenic diet only have been evaluated (50, 51). Therefore, our findings extend our understanding of the regulation of fat browning under various hyperketonemic condition. Thirdly, our results suggest that β-HB plays a role in fat browning through intracellular action, which is consistent with a previous study (18). Lastly, our results provided evidences that fat browning effects of β-HB are dependent on the AMPK-SIRT1-PGC-1α pathway, which is associated with the results of intracellular action of β-HB. However, it should be noted that further investigation is needed to confirmed the differential regulation of the AMPK-SIRT1-PGC-1α pathways and PRDM16 and UCP1 expression, according to the ketogenic diet, exercise, or combined intervention *in vivo*. This may accelerate development of pharmacological interventions combating obesity.

Our study has several limitations. First, we did not measure the real-time *in vivo* energy expenditure, which limits the interpretation of the molecular findings. Second, we could not explain the effects of whole body lipid regulation by hyperketonemic condition, as we did not measure the hepatic regulation of lipids *in vivo*. Third, our results were unable to elucidate the probable gender difference in the effects of hyperketonemia on fat browning. More importantly, did not evaluate the effects of hyperketonemia induced by ketogenic diet and/or exercise on hepatic and whole body metabolism. Therefore, although hyperketonemia induced by a ketogenic diet and/or exercise may induce fat browning, our research does not claim that this intervention is beneficial for metabolic health. Fourth, our results were unable to elucidate the mechanistic causality of β-HB-induced fat browning and adipokine expression *in vivo*. Although our *in vitro* experiments suggest the activation of fat browning pathways by intracellular β-HB, genetic manipulation of animals, such as using adipocyte-specific MCT1 knock-out mice, or conducting a loss-of-function *in vivo* experiments for fat browning-related adipokines, are necessary. Lastly, in animal experiments, plasma β-HB levels were observed to be approximately 1.5–2 mM, and direct measurements of ketone concentrations in adipose tissue were not conducted. We observed that β-HB with physiological concentration (1 ~ 2 mM) exhibited effects similar to those observed at higher concentration (4 mM) *in vitro* (see Supplementary Figures S1–S3). However, these findings should be further confirmed *in vivo* by treating animals with β-HB doses that achieve physiologically relevant concentrations. In addition, further experiments are required to ascertain whether the fat browning effect induced by a cellular level of 4 mM β-HB concentration can be representative of fat browning manifestation in the KE animal group.

## 5 Conclusion

Our study demonstrated that ketogenic diet-and/or exercise-induced hyperketonemia, particularly the elevation of β-HB, can activate fat browning through the upregulation of mitochondrial biogenesis, thermogenic factors, fat browning-related adipokines, and PRDM16 in WATs of non-obese rats. The fat browning effects of intracellular β-HB are associated with activation of AMPK-SIRT1-PGC-1α pathway. Whether upregulation of β-HB could serve as a promising strategy to increase beige fat formation and provide metabolic benefits in human requires further investigation.

## Data Availability

The datasets presented in this study can be found in online repositories. The names of the repository/repositories and accession number(s) can be found in the article/Supplementary material.
