# Supplementary material for: Ketogenic diet with aerobic exercise can induce fat browning: potential roles of β-hydroxybutyrate
Source: Front Nutr. 2024 Aug 29;11:1443483. doi: 10.3389/fnut.2024.1443483 (PMC11390540; doi:10.3389/fnut.2024.1443483)
Supplement: Supplementary file 1 [file Data_Sheet_1.pdf]

Supplementary Material

Supplementary Figures

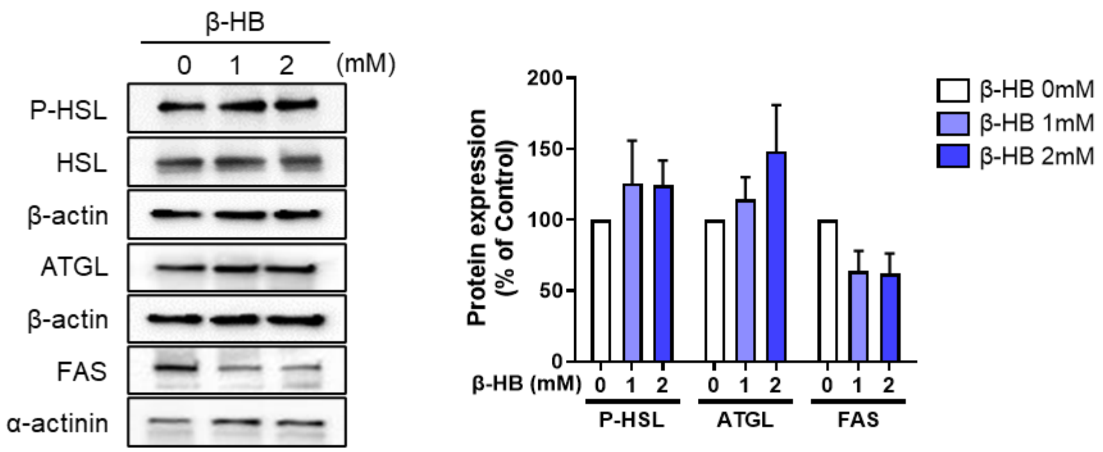

Supplementary Figure 1. Effects of β-HB with low concentration (1~2 mM) on the protein expression of lipolytic and lipogenic enzymes. During differentiation, 3T3-L1 cells were treated with b-HB with vehicle, or β-HB (n = 4).

(A)

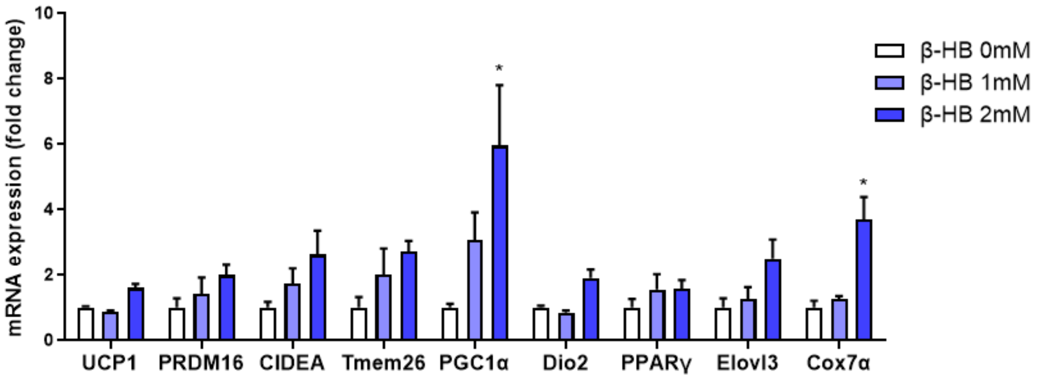

(B)

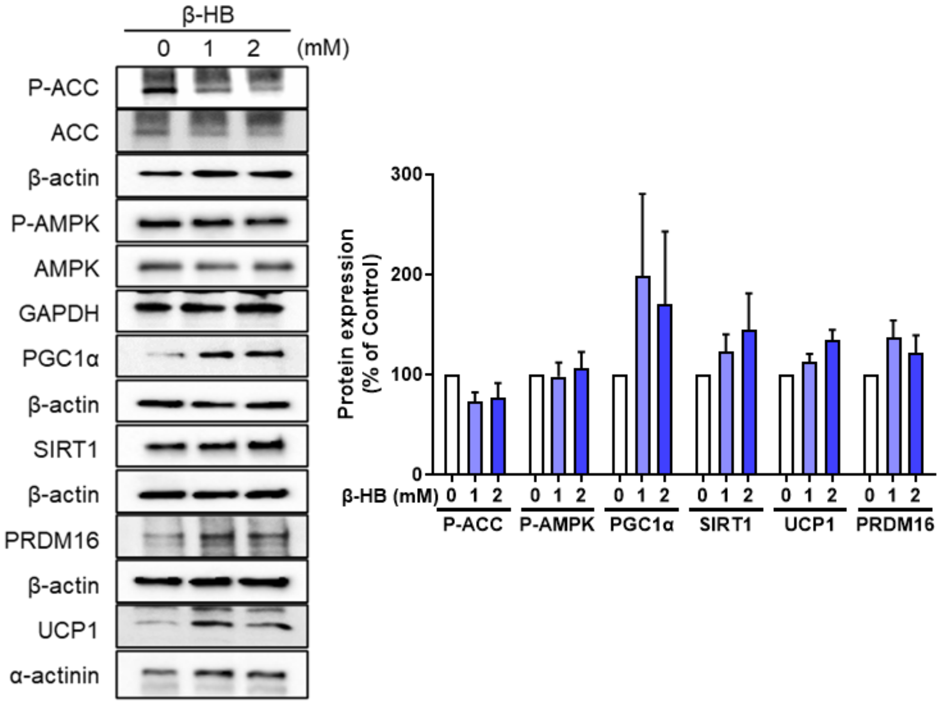

Supplementary Figure 2. Effects of β-HB with low concentration (1~2 mM) on the mRNA (A) and protein (B) expression of thermogenic and fat browning-related factors. 3T3-L1 cells were treated with vehicle or β-HB during differentiation (n = 4).

(A)

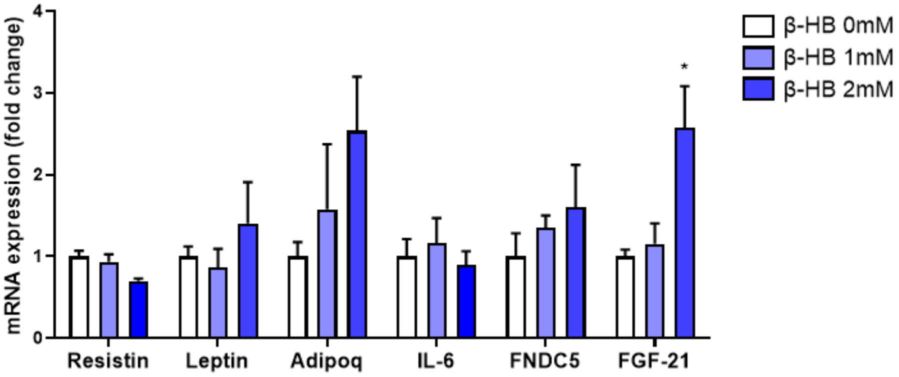

(B)

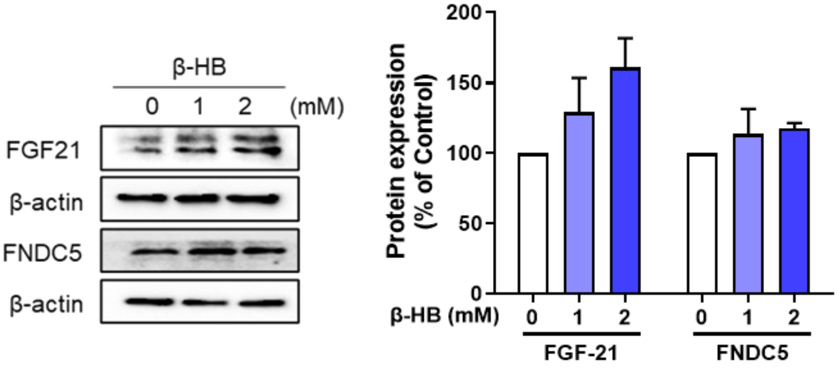

Supplementary Figure 3. Effects of β-HB with low concentration (1~2 mM) on the mRNA (A) and protein (B) expression of fat browning-related adipokines. 3T3-L1 cells were treated with vehicle or β-HB during differentiation (n = 4).

Supplementary Table S1. Primer sequences for RT-PCR

Gene		sequences
AdipoQ	F	5'- GAC GTT ACT ACA ACT GAA GAG C - 3'
	R	5'- CAT TCT TTT CCT GAT ACT GGT C - 3'
Cidea	F	5'- CTA GCA CCA AAG GCT GGT TC - 3'
	R	5'- CAC GCA GTT CCC ACA CAC TC - 3'
Cox7α1	F	5' – CAG CGT CAT GGT CAG TCT GT – 3'
	R	5' – AGA AAA CCG TGT GGC AGA GA – 3'
DIO2	F	5' – CAG TGT GGT GCA CGT CTC CAA TC – 3'
	R	5' – TGA ACC AAA GTT GAC CAC CAG – 3'
Elovl3	F	5' – TCC GCG TTC TCA TGT AGG TCT – 3'
	R	5' – GGA CCT GAT GCA ACC CTA TGA – 3'
FGF-21	F	5'- TAC ACA GAT GAC GAC CAA GA - 3'
	R	5'- GGC TTC AGA CTG GTA CAC AT - 3'
FNDC5	F	5'- ATG AAG GAG ATG GGG AGG AA - 3'
	R	5'- GCG GCA GAA GAG AGC TAT AAC A - 3'
GAPDH	F	5'- CAA CTC CCA CTC TTC CAC CT - 3'
	R	5'- CTT GCT CAG TGT CCT TGC TG - 3'
IL-6	F	5'- CAG CTA TGA ACT CCT TCT CCA C - 3'
	R	5'- GAG ATG CCG TCG AGG ATG TAC - 3'
Leptin	F	5'- CCA AAA CCC TCA TCA AGA CC - 3'
	R	5'- CTC AAA GCC ACC ACC TCT GT - 3'
PGC1α	F	5' – CCC TGC CAT TGT TAA GAC C – 3'
	R	5' – TGC TGC TGT TCC TGT TTT C – 3'
PPARγ	F	5' – AGT GTG AAT TAC AGC AAA TCT CTG TTT T – 3'
	R	5' – GCA CCA TGC TCT GGG TCA A – 3'
PRDM16	F	5'- GCA CTT GCT TAA ATA CAT ATC ACG TGT T - 3'
	R	5'- CAG CTC GGA GGC CTT TTC T - 3'
Resistin	F	5'- CCT CCT TTT CCT TTT CTT CC - 3'
	R	5'- AGG AGA CTG TCC AGC AAT TT - 3'
Tmem26	F	5' – CTG TGT TCT CAT TCT CGG CTT TG – 3'

	R	5’ – GTG CTT GGT GGC TCA TTC TTC – 3’
**UCP1**	F	5’- GAC CGA CGG CCT TTT TCA A- 3’
	R	5’- AAA GCA CAC AAA CAT GAT GAC GTT - 3’

Supplementary Table S2. The list of antibodies used for the expression of protein.

**Antibodies**	**Dilution**	**Catalog#**	**Manufacturer**
anti-phospho-ACC	1:1000	#3661	Cell Signaling Technology
anti-ACC	1:1000	#3662	Cell Signaling Technology
anti-phospho-AMPK	1:1000	#2535	Cell Signaling Technology
anti-AMPK	1:1000	#2532	Cell Signaling Technology
anti-FNDC5	1:250	ab131390	Abcam
anti-FGF21	1:500	ab64857	Abcam
anti-PGC1α	1:500	ab54481	Abcam
anti-PRDM16	1:1000	ab106410	Abcam
anti-SirT1	1:1000	#9475	Cell Signaling Technology
anti-UCP1	1:500	ab10983	Abcam
anti-ATGL	1:1000	#2138	Cell Signaling Technology
anti- phospho-HSL	1:1000	#45804	Cell Signaling Technology
anti-HSL	1:1000	#4107	Cell Signaling Technology
anti-FAS	1:1000	#3180	Cell Signaling Technology
anti-C/EBPα	1:1000	#2295	Cell Signaling Technology
anti-PPARγ	1:1000	ab209350	Abcam

anti-OXPHOS complex	1:1000	ab110413	Abcam
anti-α-actinin	1:2000	sc-166524	Santa Cruz Biotechnology
anti-β-actin	1:10000	PM-7547	Pro-sci
